# Selective Synthesis of Polyoxyethylene–Polyoxypropylene Block Copolymer (Poloxamer) Fatty Acid Monoesters Over Homogeneous Organotin Catalyst

**DOI:** 10.1007/s11743-017-2020-2

**Published:** 2017-09-26

**Authors:** Janusz Nowicki, Julia Woch, Małgorzata Mościpan, Andrzej Robaszkiewicz, Rafał Grabowski, Izabela Semeniuk, Karol Erfurt

**Affiliations:** 1 Institute of Heavy Organic Synthesis “Blachownia”, Kędzierzyn-Koźle, Poland; 2 Faculty of Chemistry, Silesian University of Technology, Gliwice, Poland

**Keywords:** Poloxamer, Esterification, Monoesters, Fatty acid, Organotin catalyst

## Abstract

The synthesis of selected polyoxyethylene–polyoxypropylene block copolymer (poloxamer) fatty acid monoesters is presented. Organotin homogeneous catalyst Sn *bis*(2-ethylhexanoate) effectively catalyzed the esterification reaction of (EO)–(PO)–(EO) block copolymer (poloxamer) with fatty acids. The reaction proceeded in high yield and high selectivity to monoesters. Content of diesters in final products was below 1 wt%. The new protocol opened up a high yield and high selective method for the synthesis of poloxamer fatty acid monoesters. These products are potentially interesting for industrial applications, e.g. in lubricants, cosmetics and, in particular, as potential emulsifying agents compatible with hydrocarbon bases, such as paraffin.

**Electronic supplementary material:**

The online version of this article (doi:10.1007/s11743-017-2020-2) contains supplementary material, which is available to authorized users.

## Introduction

Polyoxyalkylenene glycol esters are a group of nonionic surfactants with a vast field of application. Polyoxyethylene and polyoxypropylene glycol fatty acid esters, prepared by esterification of fatty acids with corresponding polyoxyalkylene glycols, are classified as non-toxic and non-irritating nonionic emulsifiers. Depending on their molecular weight and water solubility, esters can be applied in both aqueous and non-aqueous systems. Polyoxyalkylene esters of fatty acids are widely used as emulsifiers in cosmetics and textiles [1, 2]. Fatty acid polyol esters have also been recently used for removal of soil (or water) contamination caused by spilled oil. Herein, their relatively low toxicity for marine fauna plays a significant role [3, 4]. Important representatives of discussed group—laurates, stearates and oleates, primarily used as emulsifiers in cosmetics and pharmaceutics, are also applied as components for drilling and cutting oils [5]. Application as lubricants and filament cohesion agents for fiber finishing is less relevant, because of their limited solubility in water.

Polyol esters are produced by chemically catalyzed esterification [6, 7]. Fatty acid esters of polyoxyethylene glycols, in general, are prepared either via oxyethylation or esterification route [8]. For increased selectivity of monoesters, a large excess of polyoxyethylene glycol to fatty acid, even 6–12 molar ratio, is recommended [9]. After the reaction, excess of polyoxyethylene glycol is washed out with concentrated salt solution [10]. The original method of the selective synthesis of monoesters using boric acid was reported by Hartman, but this method is of little preparative significance [11]. Many of the esterification reactions are carried out at elevated temperatures in presence of homogeneous acidic, amphoteric or basic catalysts [12]. Sodium or potassium hydroxide or *p*-toluenesulphonic acid are the most preferred catalysts. In order to favorably shift the equilibrium, esterification water is removed by applying vacuum or using a flow (stream) of nitrogen.

Fatty acid esters of polyoxyethylene glycols can be also produced from fats or plant oils in transesterification processes in presence of strong basic catalysts. The reaction product in general is a mixture of mono-, di- and triglycerides, glycerol, unreacted polyoxyethylene glycol and also a mixture of mono- and diesters [13, 14]. Because of the very complex character of the product this method is not preferred.

Diesters of polyalkylene glycols can be selectively synthesized with high yields by using a high molar excess of fatty acid. Synthesis of monoesters, which are characterized by more interesting surface active properties (e.g. as emulsifier for textile industry) [15], is possible, but requires the use of high molar excess of glycol [16]. Equal activity of both hydroxyl groups in polyoxyethylene glycols leads to esterification of both of hydroxyl groups. As reported by Corma *et al*. better miscibility of fatty acids with the polyoxyalkylene glycol monoesters compared to glycol promotes the reaction of fatty acids with hydroxyl group of monoesters, rather than with polyoxyethylene glycol, which is immiscible with fatty acids. In addition to that, disproportionation of monoester formed in the first step by transesterification also leads to increased amounts of diester [17].

Fatty acid monoesters of poly(oxyalkylene) glycols can be also obtained in oxyalkylation route. Direct oxyalkylation of fatty acids with conventional basic catalysts yields a complex mixture of mono- and diesters, as well as various polyethylene glycols as by-products, with a wide range of polyethylene glycol units. The final product can be used as emulsifiers in food, cosmetics and other technical applications [18, 19].

In recent years, enzyme-catalyzed high yields synthesis of fatty acid esters and fatty acids and polyoxyalkylene glycols through esterification and interesterification processes have attracted particular attention. The catalytic activity of lipases toward hydroxy fatty acid esters was well studied by Hayes [20–23]. In the esterification of several hydroxyl fatty acids *Rhizomucor miehei* lipase was also adopted [24–27]. Steffen *et al*. used *R. miehei* and *Candida antarctica* lipases as biocatalyst for the synthesis of monoglycerides of 17-hydroxy- and 12-hydroxystearic acids with high yield [27]. A promising alternative for this processes could be the use of homogeneous organometallic Lewis compounds as catalysts, which are used in many other commercial processes, e.g. synthesis of polyesters. Our previous experiments on the esterification reaction of higher fatty acids and polyols conducted over various Ti, Zr and Sn catalysts have shown that homogeneous Sn *bis*(2-ethylhexanoate) is characterized by the highest activity and selectivity to the desired products [28].

In this paper, the novel high selective method of the synthesis of monoesters of selected hydrophobic polyoxyalkylene glycols—polyoxyethylene–polyoxypropylene block copolymers (poloxamer) is presented. In the literature, there is practically no detailed information on the synthesis of poloxamer fatty acid esters. Only in the FDA report can find information, that poloxamer fatty acid esters are safe and can be used to manufacturing materials, that come in contact with food [29]. A homogeneous Sn *bis*(2-ethylhexanoate) catalyst was used. The described method under optimal reaction conditions results in the desired monoesters with high selectivity, above 99%.

## Experimental

Oleic acid 90+% was purchased from Croda Int. as Priolene 6936. Stearic acid 99+%, lauric acid 99+% and octanoic acid 99+% were purchased from Sigma Aldrich. Polyoxyethylene–polyoxypropylene–polyoxyethylene block copolymer (poloxamer) was purchased from BASF as Pluronic PE3100 (mol mass = 1000; molar mass of propylene glycol block = 850; polyethylene glycol content = 10 wt%; hydroxyl value = 105 mg KOH/g). Commercially available homogeneous Sn *bis*(2-ethylhexanoate) purchased from PMC Organometallic as Fascat 2003 was used as catalyst. Cyclohexane and potassium phosphate analytical grade (Pure) were purchased from Sigma Aldrich.

Gel permeation chromatography (GPC) was carried out on a L-7100 series pump (Merck-Hitachi) equipped with degasser (Knauer) and three column series from Polymer Laboratories, Inc. (Amherst, MA, USA) consisting of PLgel 3 μm Guard (7.5 × 50 mm), PLgel 5 μm MiniMIX-E (7.5 × 250 mm, molecular weight range 500–30,000 g/mol) and PLgel 5 μm MiniMIX-E (7.5 × 250 mm, molecular weight range 500–30,000 g/mol) columns. The system was fitted with a VISCOTEK VE 3580 differential refractometer detector and anhydrous tetrahydrofuran was used as the mobile phase (0.3 mL min flow rate). Data were collected and processed using GRAMS/386 for Chromatography software and calibrated against polystyrene standards. Analyses were performed at 30 °C.

^1^H-NMR spectra were recorded on Varian Unity Inova Plus spectrometer (CDCl_4_, 400 MHz) FT-IR spectra were recorded on MATTSON 3000 spectrometer (Unicam) at 500–4000 cm^−1^. Kinematic viscosity was determined on Brookfield DV-II+ viscometer. Density of esters at 25 °C was determined using 1 cm^3^ micro-pycnometer. Hydrophile-lipophile balance values (HLB) of polyol esters were calculated according to Davies method [30].

### Synthesis of Polyol Monoesters (General Procedure)

In a 200 mL glass reactor, equipped with mechanical stirrer, an electronic temperature controller, glass capillary and a receiver to collect esterification water were placed 180 g of Pluronic PE3100 and the required amount of fatty acid (according to desired COOH:OH molar ratio calculated on the basis of hydroxyl value). The reactor was heated up to 150 °C and then a proper amount of catalyst was added into the reactor. Temperature was raised to 220 °C and the reaction mixture was then stirred for 9 h. Nitrogen introduced into the reactor (glass capillary) helps to remove the water and also provides a protective atmosphere. Esterification was considered complete when the acid number of the reaction mixture was below 1 mg KOH/g. Crude esters were then diluted with cyclohexane in order to decrease the viscosity and density. This solution was washed at first by 5 wt% of K_3_PO_4_ water solution and twice with deionized water to reach a neutral pH. Solvent was removed under reduced pressure on a rotary evaporator.

## Results and Discussion

It is well known that the esterification reaction is strongly influenced by the efficiency of water removal. In the case of low molecular weight alcohols (methanol, ethanol), which do not form heteroazeotropic mixtures with water, the process is difficult. In such type of alcohols the satisfactory results are achieved by using a large excess of alcohol (e.g. direct esterification of fatty acid with an excess of methanol). A more convenient situation is observed in the case of higher aliphatic alcohols (> C_4_). They form heteroazeotropic mixtures with water, which facilitates the removal of the esterification water. Unfortunately, for higher aliphatic alcohols (> C_10_) and low volatility polyols the above method is not practical. In these cases water can be removed with the use of neutral solvents (e.g. toluene) or by running the esterification reaction at elevated temperatures. This method is applied in the esterification of polyols, also polyoxyalkylene glycols, with fatty acids. However, because of relatively high melting points of polyols and their limited miscibility with fatty acids, an esterification temperature above 150 °C is required.

To obtain an efficient emulsifying agent, a higher content of monoester in the reaction product is preferred (higher hydrophilicity and higher HLB value). The choice of catalyst and reaction conditions are crucial for high selectivity of monoesters. Monoesters of polyxyalkylene glycols are characterized by better emulsification potential, especially in terms of w/o emulsions. EO/PO/EO block copolymers are a mixture of oligomers with various molecular masses characterized by different values of hydroxyl number.

The aim of this study was too selectively obtain fatty acid monoesters of EO–PO–EO block copolymer (Pluronic PE3100). To find the optimal COOH:OH molar ratio, a series of esterification reactions with different molar ratios were performed. As a control parameter, acid value of the crude ester was adopted. The catalyst (tin octanoate) did not affect the overall acid number in post-synthesis mixtures, so the acid value of the esterification product catalyzed by metalorganic Lewis catalysts depended mainly on the free fatty acid content.

**Fig. 1:**
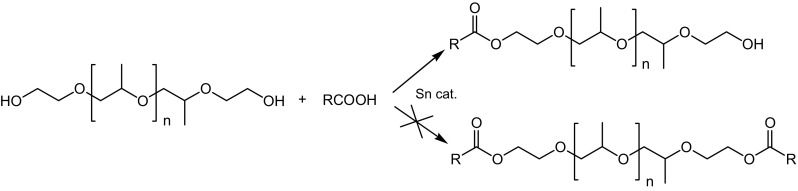
Synthesis of fatty acid EO–PO–EO block copolymer glycol monoesters

Experiments were conducted using fatty acid:OH ratio of 1:0.45–1:0.55 in the presence of Sn *bis*(2-ethylhexanoate) as a catalyst at 220 °C. The esterification reaction in the above adopted reaction conditions resulted mainly in monoesters according to the scheme presented in Fig. 1. Similarly to esterification of polyols, the molar ratio of fatty acid and OH group is the key factor affecting the composition of post-synthesis mixtures. In EO–PO–EO block copolymers, the terminal groups are primary hydroxyl groups, so this type of polyols in esterification reaction react similarly to most aliphatic α,ω-glycols. However, polyoxyalkylene glycols are a mixture of glycols with various oxyalkylene units and, in consequence, of various molar mass (Gauss rule).

The amount of added catalyst is considered as one of the important parameter in the esterification reaction. The results of preliminary studies of the esterification reaction of oleic acid and Pluronic PE3100 in relation to the amount of added Sn catalyst are presented in Table 1. The amount of added catalyst had noticeable less importance on final acid values of crude reaction product. Acid value of crude esters obtained for the amount of catalyst at the level 0.7–0.9 wt% was very similar, so in the following experiments the catalyst was added at 0.7 wt% only.

**Table 1:** The results of the esterification reaction of oleic acid and Pluronic PE3100 (catalyst amount effect)

catalyst amount, wt%	Acid value, mg KOH/g
0.5	0.98
0.7	0.54
0.9	0.50

Reaction conditions: temp.—220 °C; reaction time—9 h; COOH:OH molar ratio—0:0.45

In Table 2 are presented the results of esterification reaction of oleic acid and Pluronic PE3100 in relation to COOH:OH molar ratio. Results presented in Table 2 shows that product containing the higher amount of monoester was obtained for a molar ratio below the theoretical value (0:0.5). For the molar ratio COOH:OH = 0:0.45 the product contained almost entirely monoester. Assuming the previously established optimum reaction parameters the esterification of Pluronic PE3100 with the series of fatty acids was then conducted. The results of these experiments are presented in Table 3.

**Table 2:** The results of the esterification reaction of oleic acid and Pluronic PE3100 (reactants molar ratio effect)

COOH:OH molar ratio	Monoester, wt%	Diester, wt%
0:0.55	95.5	4.5
0:0.5	97.4	2.6
0:0.45	99.0	1.0

Reaction conditions: temp.—220 °C; time—9 h; catalyst amount—0.7 wt%

**Table 3:** Analysis of crude fatty acid Pluronic PE3100 monoesters

Fatty acid	Acid value, mg KOH/g	Monoester, wt%	Diester, wt%
Octanoic acid	0.55	99.2	0.8
Lauric acid	0.50	~ 100	< 0.5
Oleic acid	0.54	99.0	1.0
Stearic acid	0.60	~ 100	< 0.5

Reaction conditions: temp.—220 °C; reaction time—9 h; COOH:OH molar ratio—0:0.45; catalyst amount—0.7 wt%

Results presented in Table 3 shows that both the acid values and the mono/diester composition for fatty acids used are similar to oleic acid monoester. The acid value of crude products was 0.5–0.6 mg KOH/g and the content of diesters was below 1 wt%. Results presented in Table 3 clearly shows that the use of organotin catalyst in the synthesis of EO–PO–EO block copolymers allows for obtaining monoesters with high selectivity, above 99%. These results, especially the selectivity towards monoesters in the presented case, were significantly higher than results described in the literature [15]. In the cited study polyoxyethylene glycol was used as polyol. EO–PO–EO glycols contain terminal hydroxyethyl groups, so they can be considered as polyoxythylene glycols analogs.

**Fig. 2:**
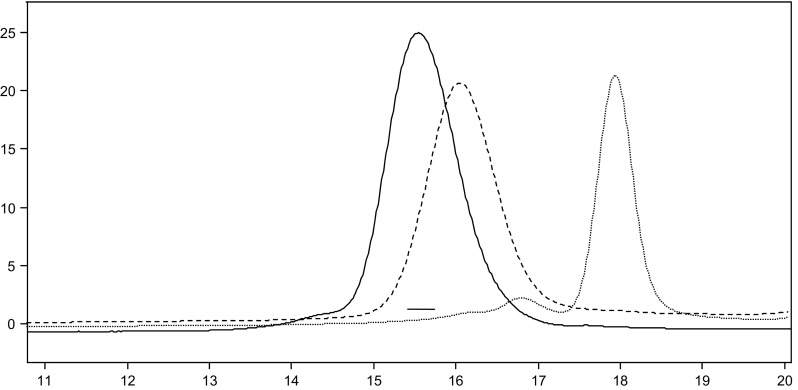
GPC chromatograms of Pluronic PE 3100 monooleate (line), Pluronic PE 3100 (dashed line) and oleic acid (dotted line)

The main analytical method widely adopted for determination of oxyalkylene fatty acid ester is gel permeation chromatography (GPC), also used in this study [31]. The results of the esterification of Pluronic PE3100 with oleic acid is presented in Fig. 2 (for GPC analysis of all esters see Supporting Information). As demonstrated in Fig. 2, the crude post-reaction mixture contains only one type of compounds (mixture of glycol monoesters). In order to confirm the contents of reaction products, the reaction mixture was compared with the raw materials used in the synthesis (polyol, fatty acids). Figure 2 clearly shows that the product of esterification consists mainly of monoesters. Small amount of diesters were detected in the case of oleic acid esterification. The lack of free oleic acid confirms high conversion rate of the fatty acid.

Crude esters were purified in order to remove the catalyst and to decrease the amount of free fatty acids in the product. Among various washing methods, the one incorporating a dilute solution of K_3_PO_4_ was adopted. The amount of basic phosphate was a three-fold molar excess relative to the acid values of the crude esters. This method was considered as the best for purification of crude fatty acid esters of various polyols [28].

Chemical structures of obtained esters were confirmed by instrumental analysis. The ^1^H-NMR spectra recorded for EO–PO–EO glycol were typical for analogous fatty acid esters. In ^1^H-NMR spectra recorded for Pluronic PE3100 fatty acid monoesters several characteristic signals (ppm): 0.88 (terminal –C**H**_**3**_); 2.30 (ester **–**C**H**_**2**_CO–); 4.22 (ester –CO–OC**H**_**2**_–) and additional 5.34 (–C**H**–C**H**–) for oleic acid ester can be found (see Supporting Information). ^1^H-NMR spectrum of polyoxyalkylene glycol oleic acid ester is very similar to another oleic acid esters [31].

In the FT-IR spectra recorded for Pluronic PE3100 fatty acid monoesters, several characteristic bonds can be found. The strong intensity OH stretching vibrations in the region of 3485–3475 cm^−1^ corresponding to terminal OH groups confirm the presence of glycol monoesters. The strong intensity bond at 1736 cm^−1^ corresponds to ester carbonyl group. The strong intensity bond at the region 1112–1109 cm^−1^ corresponds to polyol ether groups. In all FTIR spectra two characteristic bonds at 1015 and 929 cm^−1^ are observed that corresponds to oxypropylene segment (see Supporting Information).

Density of purified esters was at the level of 0.878–0.882 g/cm3. Kinematic viscosity of Pluronic PE3100 monoesters depended on the fatty acid used. Viscosity values, measured at 25 °C, ranged from 100 to 152 mPa s. Physicochemical data of Pluronic PE3100 fatty acid monoesters are presented in Table 4.

**Table 4:** Physicochemical characteristic of purified fatty acid Pluronic PE3100 monoesters

Fatty acid	Acid value, mg KOH/g	Density, g/cm^3^	Viscosity, mPa/s	HLB
Octanoic acid	0.25	0.878	100.0	7.36
Lauric acid	0.20	0.882	137.5	5.55
Oleic acid	0.24	0.879	152.5	2.70
Stearic acid	0.20	0.880	148.7	2.70

HLB values of synthesized monoesters were determined according to the modified additive Davies method. For surfactant containing CH_2_, EO, PO can be written as:

$$ {\text{HLB}} = 7+ N({\text{CH}}_{ 2} ) \times n{\text{CH}}_{ 2} + N({\text{EO}}) \times n({\text{EO}}) + N({\text{PO}}) \times n({\text{PO}}) + \varSigma {\text{ other hydrophilic group}} + \varSigma {\text{ other hydrophobic groups,}} $$

where *N*(CH_2_), *N*(EO), and *N*(PO) are the number of groups CH_2_, EO and PO. *n*(CH_2_), *n*(EO) and *n*(PO) represent the chain lengths for CH_2_, EO and PO groups. Beside of CH_2_, EO and PO groups, Pluronic monoesters contains also ester C(O)O and OH group as other hydrophilic group and terminal CH_3_ as other hydrophobic groups. Above equation for synthesized esters can be written as:

$$ {\text{HLB}} = 7+ N({\text{CH}}_{ 2} ) \times n({\text{CH}}_{ 2} ) + N({\text{EO}}) \times n({\text{EO}}) + N({\text{PO}}) \times n({\text{PO}}) + N({\text{COO}}) + N({\text{OH}}) + N({\text{CH}}_{ 3} ). $$

*N* values have been adopted from widely published data as follow: *N*(EO) = 0.33; *N*(ester) = 2.4; *N*(OH) = 1.9; *N*(PO) = −0.15; *N*(CH_*x*_) = −0.475 [30, 32]. The results of HLB calculations were collected in Table 4. According to widely adopted applications for nonionic surfactants within the HLB ranges, octanoic and lauric acid monoester of Pluronic PE3100 can be classified as W/O emulsifier and oleic and stearic acid monoester as defoamers [33].

## Conclusions

The results described in this study show that homogeneous organotin catalyst is characterized by high activity and selectivity in the synthesis of fatty acid monoesters of Pluronic PE3100 EO–PO–EO block copolymer. Sn *bis*(2-ethylhexanoate) proved to be useful in the synthesis of oxyalkylene glycols with longer fatty acid chains that are potentially suitable as W/O emulsifier and defoamers. In optimal reaction conditions polyoxyalkylene glycol fatty acid esters with high selectivity to monoesters (99%) were obtained. Simple synthesis procedure, relatively mild reaction conditions, commercial availability of catalyst and also no need for difficult and expensive purification stages enable, in our opinion, application of this method in industrial scale.

## Electronic supplementary material

Below is the link to the electronic supplementary material.

Supplementary material 1 (DOC 1055 kb)
