# Supplementary material for: Selective Synthesis of Polyoxyethylene–Polyoxypropylene Block Copolymer (Poloxamer) Fatty Acid Monoesters Over Homogeneous Organotin Catalyst
Source: J Surfactants Deterg. 2017 Sep 26;20(6):1475–81. doi: 10.1007/s11743-017-2020-2 (PMC5686269; doi:10.1007/s11743-017-2020-2)
Supplement: Supplementary file 1 — Supplementary material 1 (DOC 1055 kb) [file 11743_2017_2020_MOESM1_ESM.doc]

**Supporting Informations**

**Selective synthesis of polyoxyethylene-polyoxypropylene block copolymer (poloxamer) fatty acid monoesters over homogeneous organotin catalyst**

Janusz Nowicki, Julia Woch, Andrzej Robaszkiewicz, Małgorzata Mościpan, Izabela Semeniuk and Karol Erfunt

Institute of Heavy Organic Synthesis “Blachownia”, Energetykow 9, 47-225 Kedzierzyn-Kozle, Poland

0

5

10

15

20

25

30

5

10

15

20

a)

0

5

10

15

20

25

10

12

14

16

18

0

b)

0

10

20

30

10

12

14

16

18

20

c)

0

10

20

30

8

10

12

14

16

18

20

d)

**Figure S1.** GPC chromatograms of Pluronic PE3100 monooctanoate (a); monolaurate (b);

monooleate (c) and monostearate (d)


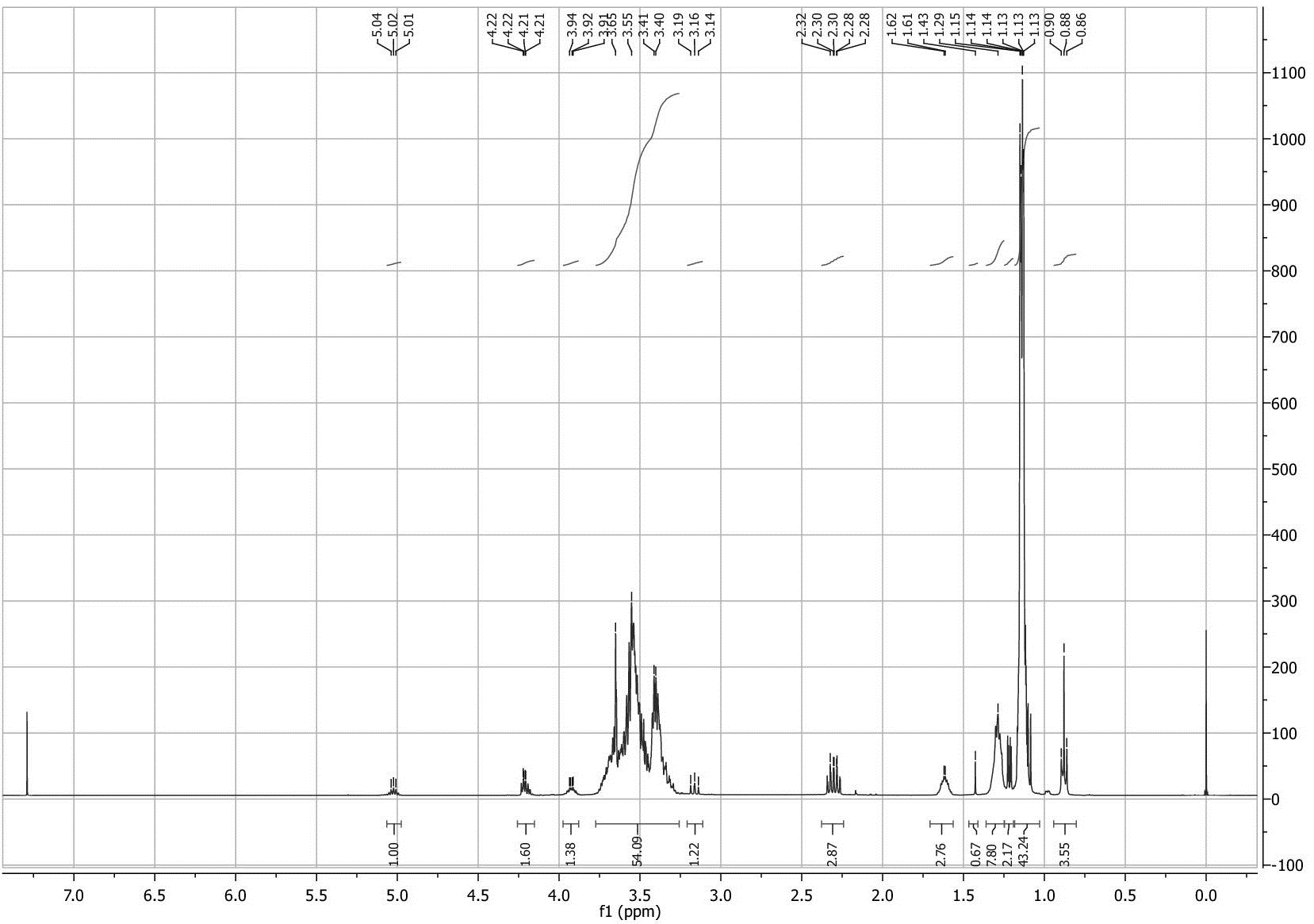


**Figure S2.** ^1^H-NMR spectrum of Pluronic PE3100 monooctanoate


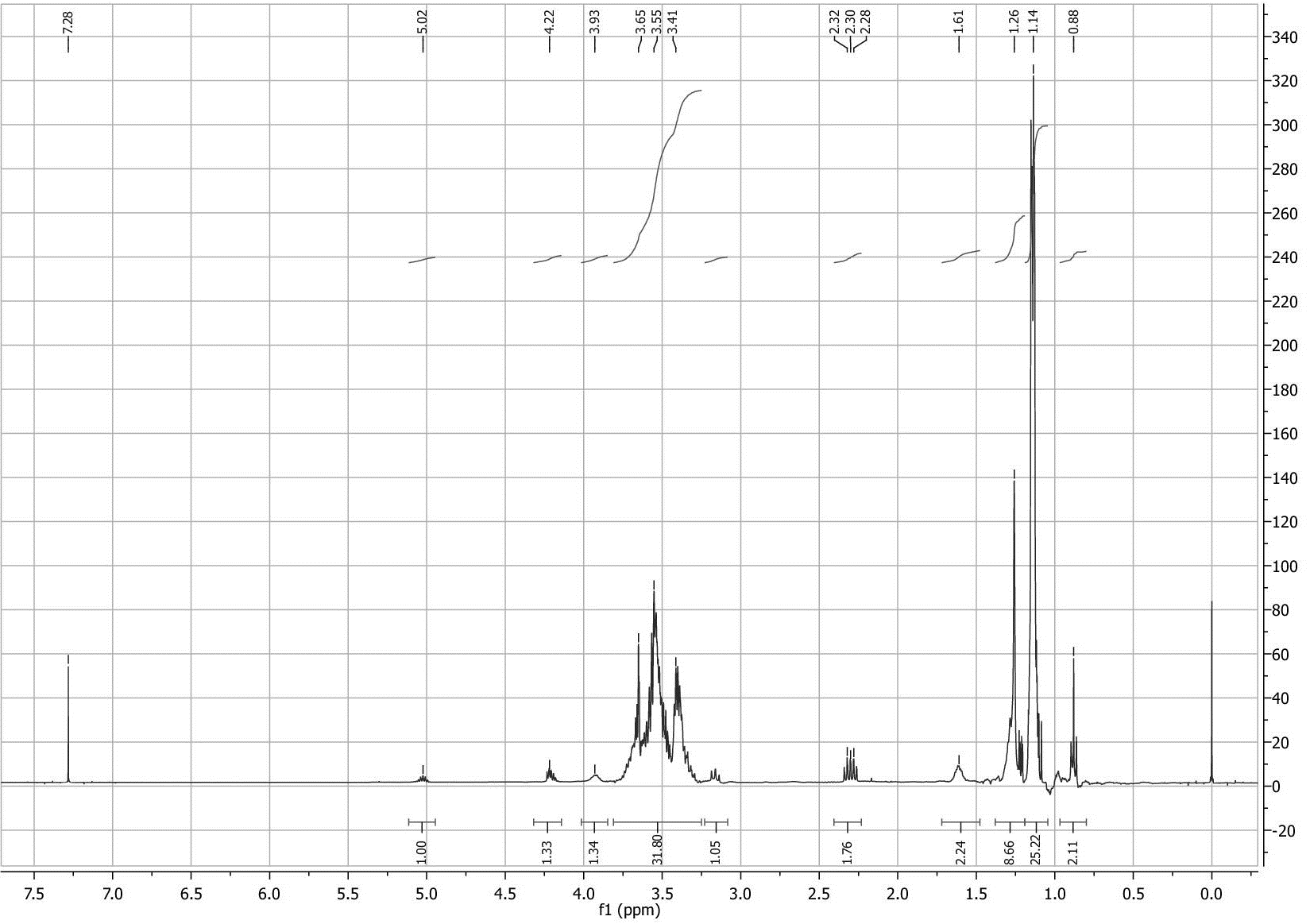


**Figure S3.** ^1^H-NMR spectrum of Pluronic PE3100 monolaurate


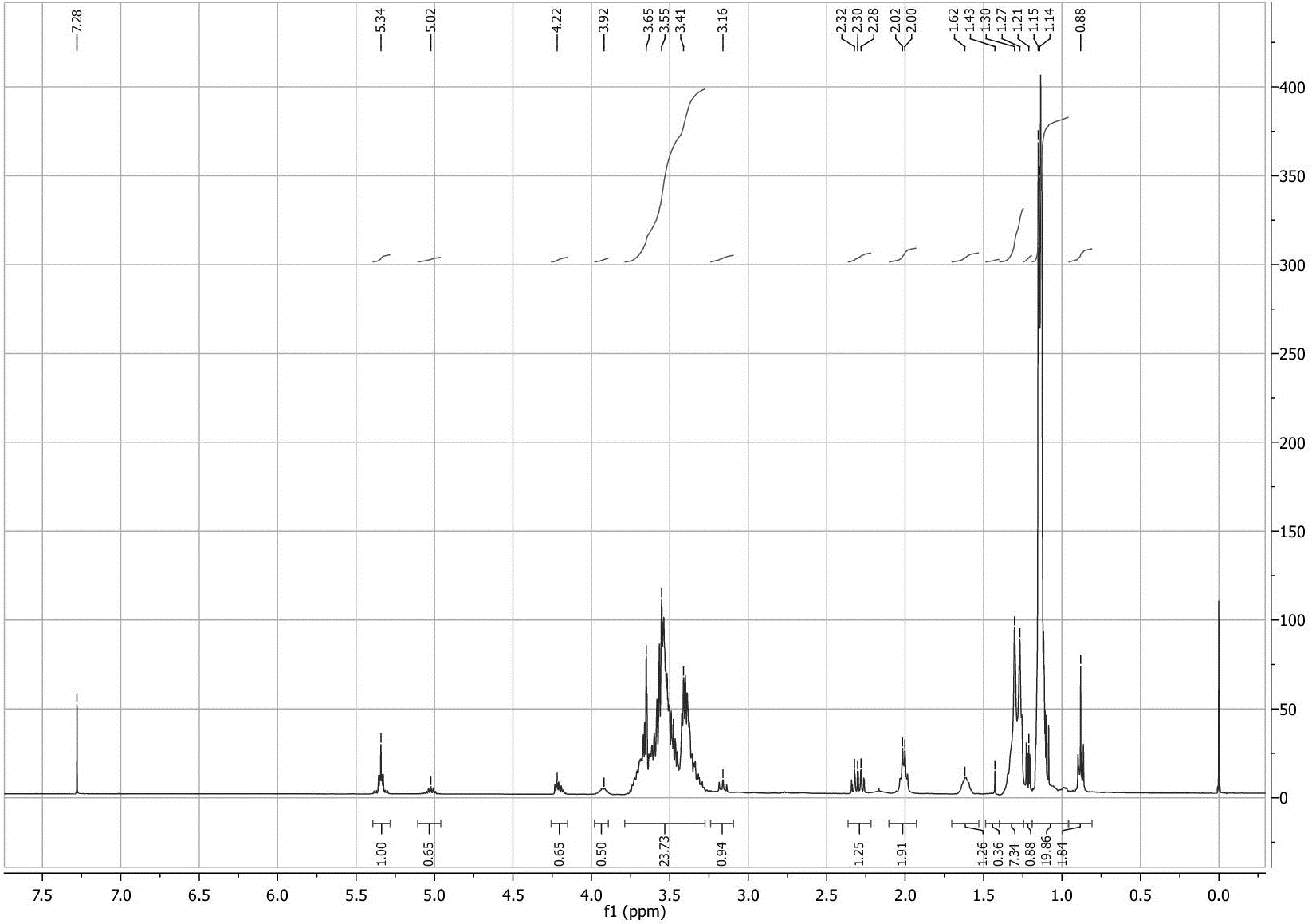


**Figure S4** ^1^H-NMR spectrum of Pluronic PE3100 monooleate


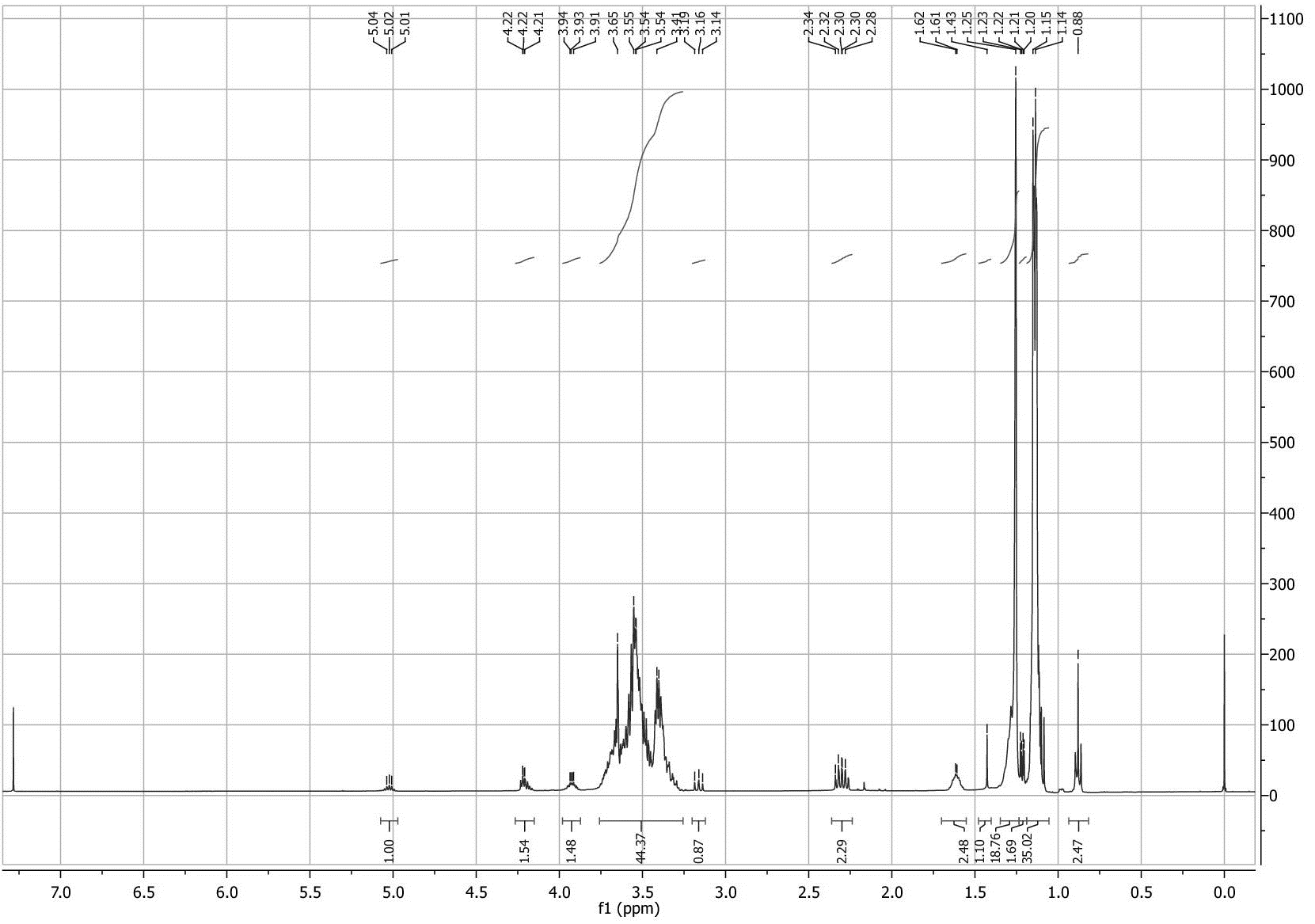


**Figure S5** ^1^H-NMR spectrum of Pluronic PE3100 monostearate

866

928

1014

1109

1256

1297

1344

1373

1455

1736

2870

2970

3478

5

10

15

20

25

30

35

40

45

50

55

60

65

70

75

80

%T

500

1000

1500

2000

2500

3000

3500

4000

(cm-1)

**Figure S6** FT-IR spectrum of Pluronic PE3100 monooctanoate

866

929

1014

1106

1256

1297

1344

1374

1457

1735

2928

2970

3483

-10

-5

0

5

10

15

20

25

30

35

40

45

50

55

60

65

70

75

80

%T

500

1000

1500

2000

2500

3000

3500

4000

(cm-1)

**Figure S7** FT-IR spectrum of Pluronic PE3100 monolaurate

866

929

1015

1109

1249

1296

1344

1373

1455

1736

2858

2927

2970

3480

-10

-5

0

5

10

15

20

25

30

35

40

45

50

55

60

65

70

75

%T

500

1000

1500

2000

2500

3000

3500

(cm-1)

4000

**Figure S8** FT-IR spectrum of Pluronic PE3100 monooleate

**Figure S9** FT-IR spectrum of Pluronic PE3100 monostearate

866

929

1014

1112

1254

1297

1344

1373

1458

1736

2857

2926

2970

3482

-10

-5

0

5

10

15

20

25

30

35

40

45

50

55

60

65

70

75

80

%T

500

1000

1500

2000

2500

3000

3500

4000

(cm-1)
